# The WEB-based Asthma Control: an intriguing connection or a dangerous hazard?

**DOI:** 10.1186/s40733-015-0017-z

**Published:** 2015-12-24

**Authors:** Carlo Lombardi, Giovanni Passalacqua, Giorgio Walter Canonica

**Affiliations:** 1 Allergy & Pneumology Departmental Unit Fondazione Poliambulanza Hospital, Via Bissolati, 57, Brescia, Italy; 2 Allergy & Respiratory Diseases, D.I.M.I., University of Genoa, Genoa, Italy

**Keywords:** Asthma control, Asthma knowledge, Patient behavior, Internet engines, Social network, Apps

## Abstract

Globally, an estimated 300 million people have asthma, presenting a considerable and increasing burden of disease for healthcare systems, families, and patients themselves. Despite two decades of guidelines, asthma seems to remain not optimally controlled in a substantial proportion of people. The achievement of asthma control is the result of the interaction among different variables concerning the disease pattern and patients’ and physicians’ knowledge and behavior. It is well known that adherence to treatment increases in parallel to patient education. There is now a growing interest in the use of digital information technologies to promote asthma control and improve outcomes. Mobile health, or mHealth, refers to mobile devices, medical sensors, and communication technologies that can enhance chronic disease care and monitoring. Aim of this review was to evaluate the web resources nowadays available and to analyze the published studies about the web-based instruments used to improve asthma knowledge, control asthma outcomes. In general, studies revealed that the technology is well accepted. Interactive asthma technology may be, in addition, of help in reaching populations difficult to reach, such as inner city populations. The number of tools and apps available continues to increase, and agencies such as the FDA, become involved in their regulation, thus the mHealth landscape will continue to evolve. Although asthma tools and apps have great potential to improve care for asthma, the proof of data reproducibility, the demonstration of effectiveness, and the privacy issues still represent the major technical problems.

## Background

Globally, an estimated 300 million people have asthma, presenting a considerable and increasing burden of disease for healthcare systems, families, and patients. Over the past 20 years, there has been a concerted effort to reduce morbidity and mortality related to chronic diseases, including bronchial asthma. These efforts led to many new initiatives to develop clinical practice guidelines, to conduct research to fill the gaps in the guidelines, and to continuously revise the guidelines themselves according to updated experimental data [1]. Despite two decades of guidelines, a substantial proportion of people do not achieve a proper asthma control. [2].

**Fig. 1:**
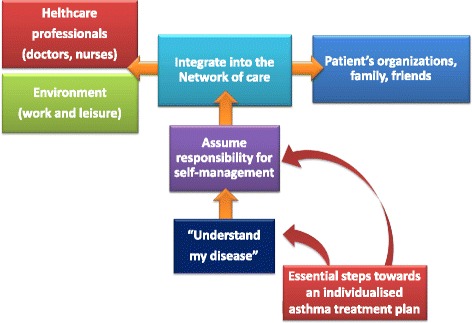
Essential steps towards an individualized asthma treatment plan

**Fig. 2:**
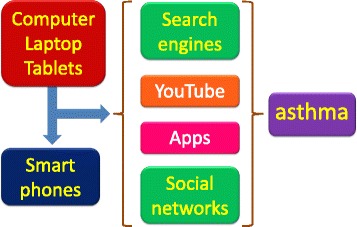
Potential network for obtain and improve information and monitoring asthma

Furthermore, the poor adherence to monitoring and treatment is a significant factor associated to the poor control of disease. In addition, the misuse of metered-dose inhalers is associated with decreased asthma stability [3]. New information about asthma phenotypes/endotypes and new tools to monitor disease activity, including biomarkers and epigenetic/“omics” markers, along with technology systems to monitor asthma control hold some promise to better identify the gaps between diagnosis and management [4]. The concept of “personalized medicine” recently emerged, and other indicators suggest that this could significantly improve asthma management, because the response to therapy is largely variable [5]. These advances should prompt the evolution of new treatments to further reduce the burden of disease. It is now imperative to continue also to focus on possibly reducing the burden of asthma and preventing its onset. The available research and surveillance show a persistent underuse of evidence-based management strategies to control asthma. The achievement of asthma control results from the interaction among different variables concerning the disease pattern and patients’ and physicians’ knowledge and behavior. The failure in asthma control thus results from the complex interaction among different variables, such as patient-related factors (i.e., adherence to treatment, alexithymia, and coping strategies) [6]. An optimal asthma control may be reached through a tailored treatment plan, taking into account the complexity of most factors that contribute to achieve and maintain this objective. Furthermore, it is known that the treatment adherence increases with increased patient education [7] (Fig. 1). Efforts in better monitoring asthma have been attempted, essentially by mean of the web-based instruments. These include electronic medical records and health care provider surveillance systems (Fig. 2). Mobile health (mHealth) refers to mobile-based monitoring systems, including medical sensors, and recording technologies that can enhance chronic disease care [8]. Combining electronic medical records with periodic measurements of pulmonary function may improve the quality of monitoring disease activity and progression [9, 10].

Web-based technologies to promote asthma control and improve outcomes are currently studied and welcome. On the other hand, there is increasing evidence that an improper and “addictive” use of the internet-based resource (such as social networks) could have a negative impact on asthma, either in term of control and self-perception. The aim of this review was to evaluate the web resources offered, and to analyze the published studies about the interactive internet-based and mobile device platforms to improve asthma knowledge, and/or outcomes, and to highlight also the possible negative effects of web-based instruments. These studies are creative and promising, but still at an early stage and, unfortunately, characterized by small trials, showing variable results [11].

## Landscape and evolution of online searches related to the medical world

Today, health information runs on the web. In the Western world it has been observed that patients spend more time online than at the doctor’s office to acquire health information. One third of the American adults go online to get information on a medical condition [12]. In the United States it is estimated that patients undergo, on average, three visits per year, but spend more than 52 h online to get health information and medical advice. 59 % of U.S. adults have looked online for health information in the past year and 35 % of adults report they have used internet for medical purposes (“online diagnosers”). 53 % of online diagnosed spoke with a clinician about what they found online and 41 % of online diagnosers had their condition confirmed by a clinician [12]. Fifty-two percent of smart-phone owners have looked up health information on their phone, compared with a 6 % of other cell phone owners. 77 % of online health seekers say they began at a search engine such as Google, Bing, or Yahoo [12]. In Italy, a Censis research within the Forum for Biomedical Research found that 32.4 % of Italians use Internet to obtain health information; out of them 90.4 % search about specific diseases, 58.6 % look for doctors and facilities and However the internet has not undermined the family doctor, who still remains the primary reference [13].

## The impact of Social Networks on asthma/asthma control

Several studies and expert opinions suggest that use of internet-based media in general might have a beneficial effect by enhancing communication, social connection, and even technical skills [14]. The dramatic increase in social network use, especially among adolescents and young adults, is leading health care researchers to further evaluate the effects of this new medium on the general population and on people affected by chronic diseases. In particular, internet-based resources can offer a patient-friendly way to monitor a gven disease and to improve adherence to treatment. At the moment, the possible negative effects, seem to be counterbalanced by increasing evidence of a positive effect on self-esteem [15]. On the other hand, Internet use was thought to promote negative psychosocial well-being, including depression and loneliness. Via social networks adolescents can come into contact with inappropriate contents, being unaware of online privacy issues. Other problems include internet addiction and concurrent sleep deprivation, as well as physical inactivity, which is associated with an increased risk of obesity [16]. Researchers recently proposed a new type of depression, termed “Facebook depression” which develops when preteens and teens spend long periods on social network sites and then begin to experience symptoms of depression [17]. In 2010, the first case of asthma exacerbation possibly triggered by the use of Facebook was reported, and other cases concerning asthma were described [18]. Social networks in general could be a new source of psychological stress and trigger exacerbations in depressed asthmatic individuals. Therefore, this type of trigger should be also taken into account when assessing asthma exacerbations. Recent studies emphasized that the effect of stressors is associated with an independent risk of developing asthma [19]. Stressful events can trigger asthma exacerbations, but could also contribute to the development of incident asthma. However, only few studies have investigated the association between stressful events and adult asthma prospectively [20]. Likewise, stress-related personality traits (e.g., neuroticism and extraversion) may increase asthma risk [21]. Psychological conditions such as anxiety and depression have been frequently reported in people with asthma and may, often associated with a poorer control [22, 23]. The hypothesis explaining this observation is that stressful life events may alter the psychological, immunological, and endocrine systems via mechanisms that are still largely unknown [24]. In fact, asthma is a chronic inflammatory disease of the airways, and many studies have linked stress and emotions to inflammatory processes [25–27]. Mechanisms of airway constriction (immune/inflammatory and cholinergic/vagal) point to two possible psychobiologic pathways: (1) psycho-neuroimmunologic, and 2) psychophysiologic (autonomic nervous system). There are evidences supporting the role of immune/inflammatory processes, involving the cholinergic system, as psychobiologic pathways [28]. Studies also support the autonomic system involvement as impacting on stress and depression in asthma [29, 30].

## The evolution of mobile Apps for asthma/asthma control

The number of medical apps greatly increased over the past decade. As of 2012, there were more than 13,000 health care-related apps available to Apple iPhone users and more than 6,000 available in the Android store [31]. Nowadays, the interest in mobile apps to manage chronic conditions such as asthma is matched by the recognition of the importance of quality and safety of apps intended for patients’ use. Changes in mobile devices and software have been accompanied by discussions that have progressively involved clinicians [32, 33], policy groups [34], and regulatory authorities [35]. There is no accepted measure of app quality, and most apps are not regulated or approved by the Federal Drug Administration (FDA), European Medical Agency (EMA) or guidelines. Mobile medical apps that the FDA would regulate include apps that transform a mobile platform into a regulated medical device; connect to an existing device for purposes of controlling its operation, function, energy source, and data transfer and storing [36]. In order for medical doctors to recommend apps to their patients, efficacy and effectiveness evaluations are necessary; however, few such evaluations are currently available. A Cochrane review of smartphone and tablet self-management apps for asthma in 2013 found only 2 trials of phone-based asthma self-management interventions that included randomized controlled trials to assess for efficacy [37]. The Authors deemed the utility of apps to be inconclusive and were unable to advise clinicians on the efficacy on apps for asthma self-management. Recently, Chen Wu et al. evaluated the currently available categories of asthma-related apps, grouped according to specific functions by the two most popular smartphone brands (Apple iPhone and Google Android), which at February 2014, comprised more than 90 % of the U.S. smartphone market [38]. Of 209 total asthma-related apps, 50 % were available for Android, 34 % for iPhone, and 16 % for both devices. Up to 58 % of apps were free and 39 % were paid. The cost of apps ranges from $0.99 to $29.99, and some (3 %) had free but less comprehensive versions available. The largest proportion of asthma-related apps (52 %) focus on teaching and training in techniques related to managing asthma. Most of these apps (39 %) teach users alternative methods for treating asthma, such as yoga postures, acupressure, and breathing exercises. Many apps (22 %) provide general information about asthma through text, video, and/or audio formats. Other apps (17 %) provide treatment information, and some explain inhalation techniques. Approximately 18 % of apps are designed for health care providers rather than for patients. Systematic analysis on all the apps identified by the Authors, however, evidenced some limitations about measurements of quality, effectiveness, and explanations of privacy risks. The same conclusions were reported by Huckvale et al. [39]. These Authors reported that: 1) between 2011 and 2013, the number of asthma apps increased from 93 to 191, despite withdrawal of 25 % of existing apps; 2) the newer apps were no more likely than those available in 2011 to include comprehensive information; 3) 13 % of all apps, were dedicated to the management of acute asthma, and recommended procedures unsupported by evidence; 4) despite the increase of apps targeting specific skills, such as acute asthma management (12 to 23) and inhaler technique (from 2 to 12), the proportion consistent with guidelines (17 %) and inhaler instructions (25 %), respectively, was low, and most apps provided only basic information about asthma (50 %) or simple diary functions (24 %). In addition, under a methodological viewpoint it was shown that theory-based web interventions works better than those non theory-based [40]. In conclusion, as the number of apps made available continues to increase, the mHealth landscape will continue to change. Although apps have great potential to improve care for asthma, most of them are currently limited in their quality, effectiveness, and protection of user data. In other words, it is unclear if substantial clinical benefits can be obtained in a landscape dominated by low-quality generic information apps.

## Asthma control and electronic asthma action plan tools (eAAP)

An asthma action plan (AAP) is a tool designed to assist asthmatic patients with self-management of their disease. Asthma guidelines recommend that all patients with asthma be provided with a plan that includes instructions for daily management and for recognizing and handling exacerbations [41]. AAPs are particularly helpful for patients with poorly controlled asthma. Some studies demonstrate that asthmatics receiving an AAP as part of their self-management education have higher satisfaction with their care, increased medication adherence, and fewer acute care visits compared with patients with no AAP [42–44]. Furthermore, a Cochrane review of 36 studies showed significant reductions in both Emergency Department visits (relative risk, 0.82; 95 % confidence interval [CI], 0.73–0.94) and hospitalizations (relative risk, 0.64; 95 % CI, 0.50–0.82) among patients with an AAP as part of optimal self-management compared with usual care [45]. Asthma action plans assist patients with self-management, but provider compliance with this recommendation is limited in part because of guideline complexity. The complexity of asthma management require also innovative approaches to improve quality gaps and patient outcomes. Technology can be leveraged to link and filter the guidelines to providers at the point of care, resulting in increased adherence and reduced exacerbations. By incorporating technology into providers’ asthma workflow, these solutions may increase the likelihood of patients receiving guideline-based recommendations and an AAP, thus facilitating their active involvement in their own care. Recently, Kuhn et al. developed an electronic AAP decision support tool (eAAP) within the electronic health record (EHR) that could streamline the evidence-based NHLBI guidelines for providers, while also creating an individualized handout for patients to be used as self-management plan [46]. The eAAP was developed in 4 phases. Phase 1 involved the creation of a web-based eAAP prototype by researchers from the General Practitioners. This prototype facilitated the ability to make design revisions quick and easy. To embed this tool into the electronic health record, a multidisciplinary team was engaged in phase 2. Once ready for production, the eAAP entered phase 3, where it was piloted at 5 outpatient primary care practices from December 2012 through July 2013. During this pilot stage, the development team continued to enhance the eAAP based on feedback from end users. Finally, in August 2013, the eAAP entered phase 4: dissemination with widespread implementation at over 100 primary care practices across the health care system. The final eAAP product meets the general criteria of typical AAPs in that it instructs patients in how to manage asthma on a daily basis and what to do in of the case of worsening symptoms or exacerbations. In addition, the eAAP provides instantaneous determination of peak flow thresholds calculated using an algorithm based on patient demographic. Between December 2012 and September 2014, 5174 patients with asthma received eAAPs. The total number of eAAP recipients re-presents approximately 10 % of the health care system’s asthma population. Compared with the controls (eAAP nonrecipients), eAAP recipients did not differ significantly in the odds of ED visits or hospitalizations for asthma at 3, 6, or 12 months. Children receiving an eAAP, however, had 33 % lower odds of receiving an oral steroid for asthma (12-month odds ratio [OR], 0.67; 95 % CI, 0.56–0.81). The combined exacerbation outcome was also significant (12-month OR, 0.73; 95 % CI, 0.61–0.87). Equivalent pre/post measures for adults were not statistically significant at 6 or 12 months, likely because of the smaller sample size; however, a 41 % decrease in outpatient oral steroids (*P* < .001) and a 34 % reduction in any exacerbation (*P* < .05) occurred 3 months after receiving an eAAP. This eAAP not only satisfies the traditional elements of basic AAPs but also leverages technology to improve the efficiency of care delivery and adherence to evidence-based guidelines with decision support capabilities to improve asthma control. This study supports existing evidence that patient self-management plays an important role in reducing asthma exacerbations.

## Conclusions

Internet, social networks, and Apps quickly are becoming universal in modern medical practice. Data sharing, online reviews and ratings, and digital privacy concerns likely will become a part of most every physician’s practice, regard-less of his or her use of social networks. The widespread use of internet-based media brings unprecedented connectivity that opens new horizons for physicians, ranging from interactions with patients, to communication with peers and the public, to novel approaches to research. This review of published clinical trials and other studies using the Internet and mobile devices to promote asthma education and improve asthma outcomes/control reveals innovation and promise. However, despite the appeal of technology-based asthma interventions, most of these studies are still in the early pilot stages with small sample sizes and mixed results. There were no studies found using currently commercially available asthma mobile device applications or social networking health sites. In general, studies revealed that the technology was well received [47–52]. Interactive asthma technology may be especially helpful in reaching populations previously considered to be difficult to reach, such as inner city populations [48–54] and those with more severe or poorly controlled asthma [55–57]. Patients with well-controlled asthma were less likely to use the technology [54]. Most studies were short-term and those that lasted longer tended to show a decline in use over time [48-55].

It is controversial whether or not these information technologies will assist persons to self-manage their asthma or completely take overall decision making. A promising approach may be to combine the use of information technology with coping peer support. Innovation in asthma information technology-based interventions requires a multidisciplinary approach among asthma doctors, behavior change specialists, health communication experts, and computer science sophistication. Other health professionals that may be an important resource to involve patients in the use of WEB in the management of asthma may be represented by pharmacists. It should be stressed, however, that the enthusiasm for using information technologies to promote asthma control must be supported by randomized trials to determine efficacy and cost effectiveness. Randomized, controlled trials with larger and more diverse sample sizes, objectively measured medical and behavioral outcomes, long-term follow-up, evaluation of cost effectiveness, and assessment of the technical feasibility are undoubtedly in the near future. The results of such studies are urgently needed. The ease with which these technologies fit into our daily lives, and the importance of ongoing patient management of chronic diseases suggest that these creative and innovative approaches could prove to be a valuable adjunct to effective clinical practice.
